# Scientists' Warning on the Rapid Evolution of Parasites in the Anthropocene

**DOI:** 10.1111/eva.70244

**Published:** 2026-04-28

**Authors:** Robert Poulin, Jerusha Bennett, Isabel Blasco‐Costa, Daniela de Angeli Dutra, Jean‐François Doherty, Eddy Dowle, Antoine Filion, Brian L. Fredensborg, Ryota Hasegawa, Kristin K. Herrmann, Devon B. Keeney, Anson V. Koehler, Janet Koprivnikar, Clément Lagrue, Henry S. Lane, Tommy L. F. Leung, Chen‐Hua Li, Colin D. MacLeod, Kim N. Mouritsen, Chris N. Niebuhr, Katie O'Dwyer, Eunji Park, Rachel A. Paterson, Bronwen Presswell, Haseeb S. Randhawa, Brandon Ruehle, Amandine Sabadel, Priscila M. Salloum, David W. Thieltges, Frédéric Thomas, Christian Selbach

**Affiliations:** ^1^ Department of Zoology University of Otago Dunedin New Zealand; ^2^ Department of Invertebrates Natural History Museum of Geneva Geneva Switzerland; ^3^ School of Environmental & Natural Sciences Bangor University Bangor UK; ^4^ Department of Zoology The University of British Columbia Vancouver British Columbia Canada; ^5^ Michael Smith Laboratories, Department of Biochemistry & Molecular Biology The University of British Columbia Vancouver British Columbia Canada; ^6^ New Zealand Institute for Bioeconomy Science Limited Lincoln New Zealand; ^7^ Department of Conservation Wellington New Zealand; ^8^ Department of Plant and Environmental Science University of Copenhagen Copenhagen Denmark; ^9^ Usujiri Fisheries Station, Field Science Center for Northern Biosphere Hokkaido University Hakodate Japan; ^10^ Department of Biological Sciences Tarleton State University Stephenville Texas USA; ^11^ Department of Biological and Environmental Sciences Le Moyne College Syracuse New York USA; ^12^ Melbourne Veterinary School University of Melbourne Parkville Victoria Australia; ^13^ Department of Chemistry & Biology Toronto Metropolitan University Toronto Ontario Canada; ^14^ New Zealand Institute of Earth Sciences Wellington New Zealand; ^15^ Zoology, School of Environmental and Rural Science University of New England Armidale New South Wales Australia; ^16^ Department of Biology University of Victoria Victoria British Columbia Canada; ^17^ Department of Biology Aarhus University Aarhus Denmark; ^18^ Marine and Freshwater Research Centre Atlantic Technological University Galway Ireland; ^19^ Department of Molecular Genetics University of Toronto Toronto Ontario Canada; ^20^ Norwegian Institute for Nature Research (NINA) Trondheim Norway; ^21^ Faculty of Life and Environmental Sciences University of Iceland Reykjavík Iceland; ^22^ New Brunswick Museum Saint John New Brunswick Canada; ^23^ Department of Natural Sciences Peru State College Peru Nebraska USA; ^24^ School of Science Auckland University of Technology Auckland New Zealand; ^25^ School of Biological Sciences University of Auckland Auckland New Zealand; ^26^ Department of Coastal Systems NIOZ Royal Netherlands Institute for Sea Research Den Burg the Netherlands; ^27^ Groningen Institute for Evolutionary Life‐Sciences University of Groningen Groningen the Netherlands; ^28^ CREEC/CANECEV, MIVEGEC (CREES) University of Montpellier, CNRS, IRD Montpellier France; ^29^ Department of Arctic and Marine Biology UiT The Arctic University of Norway Tromsø Norway; ^30^ Water Research Group, Unit for Environmental Sciences and Management North‐West University Potchefstroom South Africa

**Keywords:** anthropogenic pressures, aquaculture, climate change, food production, genomic signature, mitigation, monitoring, space‐for‐time comparisons, thermal performance curves

## Abstract

Human activities are changing the natural world at an accelerating pace, and as a consequence exerting novel and often strong selection pressures on living organisms. For species with traits conferring huge inherent evolutionary potential, like parasites, the outcome may be rapid adaptive responses spanning multiple phenotypic traits. The rise of drug resistance in parasites of domesticated animals is well documented; however, rapid changes in other key parasite traits may go unnoticed. In this contribution to the Scientists' Warning series, we argue that parasites are capable of evolving quickly to meet the new pressures of the Anthropocene. After summarizing evidence demonstrating their ability to evolve quickly and the magnitude of the anthropogenic selection pressures they now face, we discuss the basic types of adaptive responses we might expect. Next, we propose methods to track rapid parasite evolution in real time, as well as possible approaches to either slow it down or mitigate its impact on animal production systems. Our aim is to raise awareness of this concerning but underappreciated phenomenon and appeal for greater research into rapid parasite evolution in the Anthropocene and its consequences.

## 1 Introduction

We live under unprecedented conditions of our own making. The Anthropocene may not be formally recognized as a new geological epoch (Witze 2024), but it is no less real for the living world. Although its exact start date is a matter of debate, with proposals ranging from the advent of the Industrial Revolution to the mid‐twentieth century, we are now well and truly into a period of significant human impacts on the Earth's ecosystems (Zalasiewicz et al. 2011; Lewis and Maslin 2015; Steffen 2021). A key feature of this new period is the “Great Acceleration” in human activities, from population growth and tourism to energy use and industrial production, often captured in dramatic graphs characterized by exponentially rising curves as a function of time (Steffen et al. 2015). The impacts on environments and biota have been well documented, including habitat destruction, global climate change, pollution, and biodiversity loss (Grimm et al. 2013; Young et al. 2016; He and Silliman 2019; Ceballos et al. 2020; Wagner et al. 2021; Exposito‐Alonso et al. 2022).

These impacts extend beyond the dynamics of populations and communities in ecological time; they also affect organisms in evolutionary time. In some cases, such as domesticated species subjected to selective breeding and synanthropic species that live in close association with humans around the world, the evolutionary landscape has changed well before industrialization. In contrast, current anthropogenic activities exert strong selective pressures on all living organisms and have already resulted in measurable evolutionary changes (Palumbi 2001; Hendry et al. 2017; Pelletier and Coltman 2018; Catullo et al. 2019; Baltazar‐Soares et al. 2021). The textbook example of industrial melanism, involving changes in wing color in British populations of peppered moth, *Biston betularia* , was only the start. Driven by frequency‐dependent selection from avian predators, alleles associated with dark wings spread quickly as pollution darkened the tree trunks on which the moths spend the day (Cook and Saccheri 2013). Other examples of evolutionary changes occurring in just a few generations include reductions in age at maturity and adult body sizes in fish populations under intensive fishing (Law 2007; Allendorf and Hard 2009; Therkildsen et al. 2019), the decrease in horn sizes of male bighorn sheep in response to selective targeting by trophy hunters (Coltman et al. 2003), and shifts in color mimicry in stoneflies following deforestation (Ni et al. 2025).

More concerning, human‐induced evolution is coming back to haunt us with a vengeance. The widespread use of insecticides against agricultural pests has quickly been followed by the spread of insecticide resistance in multiple insect species, threatening the sustainability of crop yields (Mallet 1989; Hawkins et al. 2018). Similarly, and predictably, the indiscriminate use of antibiotics and anthelmintics has resulted, respectively, in the evolution of superbugs, that is, pathogenic bacteria resistant to most of our chemical arsenal against them (Davies and Davies 2010; Bottery et al. 2021), and parasitic worms in livestock no longer susceptible to treatment (Kaplan 2004; Sangster et al. 2018). The impact of anthropogenic pressures is not limited to pathogens and parasites of humans and domesticated animals, they may also drive evolutionary changes in those infecting wild animals (Poulin et al. 2024).

Our focus here is on parasites in general, and more specifically on traits that usually fly under the radar. While most attention has been directed at resistance in the face of continued drug treatment, multiple other traits of parasites, ranging from reproductive strategies to virulence, are also under new and intense selection. Any sustained modification to the conditions under which parasites are transmitted and exploit their hosts that affect their fitness will select for adaptive adjustments. Multiple anthropogenic activities affect the survival of parasite infective stages outside their host, their transmission success to hosts, their lifespan within hosts, or their reproductive rate. All of these select for changes to parasite life history traits. Earlier reviews drew attention to the novel sources of selection acting on parasites in the Anthropocene (Lebarbenchon et al. 2008; Mennerat et al. 2010; Cable et al. 2017; Poulin et al. 2024). Here, we want to emphasize the speed at which parasites are evolving and raise awareness of the many ways in which parasites are fast changing in response to what we do. More importantly, beyond simply sounding the alarm, we go one step beyond and offer some of the first possible solutions for the mitigation of this rapid evolution and its consequences. We first provide evidence that parasite evolution can be very rapid, and that the selective pressures exerted by human activities have significant fitness impacts for parasites. We then discuss the types of evolutionary changes we might expect in parasite populations as the Anthropocene progresses. Finally, we end by summarizing various approaches to monitor and quantify these changes, and we propose a few strategies to limit the magnitude and consequences of rapid parasite evolution. Our goal is to draw attention to this underappreciated phenomenon, highlight the threats it poses, and encourage further efforts to consider what we might do about it.

## 2 Parasites Can Evolve Rapidly

Generally speaking, in addition to being driven by an arms race with their coevolving hosts, parasites possess two key traits that can facilitate rapid evolution: short generation times combined with high fecundity relative to their body size. In some parasites, such as trematodes, both genomic architecture (Cwiklinski et al. 2015) and standing genetic variability within populations (Koehler et al. 2011; Berkhout et al. 2014) also provide the raw material for rapid evolution. The response of parasite traits to directional selection depends not only on their contribution to fitness but also on the genetic mechanisms underpinning their expression and their heritability. Qualitative traits, such as anthelmintic resistance, often depend on one or very few genes, whereas quantitative traits such as body size and other life history traits are determined by the small but additive effects of many genes (Falconer and Mackay 1996; Maynard Smith 1998). Although specific information for parasites is generally lacking, heritability of traits, that is, how much of their variation within a population is explained by genetic differences rather than environmental effects, is generally greater for qualitative than quantitative traits (Mousseau and Roff 1987). Nevertheless, under strong enough selection, we might expect any trait to respond quickly.

Unplanned, “natural” experiments gone wrong, such as the regular and continued use of anthelmintic drugs against helminths (Kaplan 2004; Sangster et al. 2018), have clearly demonstrated the evolutionary potential of parasites. There are at least two other sources of evidence that parasite traits other than drug resistance can evolve very rapidly under new selective pressures. First, controlled experiments in which parasite populations are subjected to particular selection pressures, or in which certain traits are preferentially selected by the researchers, provide estimates of the speed of evolution (Kawecki et al. 2012). Serial passage experiments have been particularly revealing (Ebert 1998). In these studies, researchers manually transmit parasites from host to host over several generations. Typically, the virulence of parasites, that is, the rate at which they exploit host resources and the associated reduction in host fitness, increases rapidly, often after fewer than 10 passages or parasite generations. The evolution of virulence is constrained by a trade‐off: higher virulence results in a greater rate of parasite reproduction, but shortens host lifespan and thus the window of time over which the parasite can transmit (Franz et al. 2025). Transmission ensured by the researchers uncouples parasite virulence from host survival, allowing the more virulent parasite genotypes to prosper. Experimental evolution studies have demonstrated that multiple other parasite traits can also respond very quickly to novel and sustained selection regimes (Table 1). To a certain extent, these rapid shifts in mean trait values in experimental parasite populations could reflect phenotypic plasticity or epigenetic processes rather than genetic changes. However, the former are often the precursor of the latter (Pfennig et al. 2010), and therefore the distinction may not matter in this context. Interestingly, selection experiments over 20 or more generations often fail to modify the original host preference of parasites and/or their performance on novel hosts (Jaenike and Dombeck 1998; Gemmill et al. 2000; Lievens et al. 2020), suggesting that host specificity may show less heritable variation than other traits.

**TABLE 1:** Selected examples of rapid parasite evolution under artificial selective pressure, along with the number of parasite generations until a significant change in trait values was observed.

Species	Host	Selective pressure	Adaptive response	Number of generations	References
*Plasmodium chabaudi* (apicomplexan)	Mice	Serial passage (maximal transmission success)	Rate of gametocyte production	11	Mackinnon and Read (1999)
*Brachiola algerae* (microsporidian)	Mosquito	Mixture vs. alteration of host lineages	Increased infection success on specific host lineages (specialization)	13	Legros and Koella (2010)
*Schistocephalus solidus* (cestode)	Crustacean	Differential survival based on growth rate	Within‐host development rate	4	Benesh (2023)
*Schistocephalus solidus* (cestode)	Crustacean	Greater survival of host manipulators	Stronger host behavioral alteration	3	Hafer‐Hahmann (2019)
*Heligmosomoides bakeri* (nematode)	Mice	Rapid passage through hosts	Growth rate, egg production, egg hatchability	8	Chehresa et al. (1997)
*Steinernema glaseri* (nematode)	Insect	Novel host	Reproductive potential	12	Stuart and Gaugler (1996)
*Columbicola columbae* (feather louse)	Bird	Preening intensity	Increased cryptic coloration	< 30	Bush et al. (2019)
*Columbicola columbae* (feather louse)	Bird	Changing host body size	Altered parasite body size (leading to reproductive isolation)	60	Villa et al. (2019)
*Callosobruchus maculatus* (seed beetle)	Plant seeds	Host switch from small to large host	Altered larval competitiveness, egg size, fecundity	36	Fox and Messina (2018)

Second, the long‐term maintenance of parasite populations in laboratory cultures, closed to immigration of new individuals and kept under artificial conditions to serve as a ready source of experimental material, can also lead to rapid and usually unwanted evolutionary changes due to altered selective pressures and reduced genetic variation. This manifests as genetic and/or phenotypic divergence between wild and laboratory populations, or among different laboratory populations maintained under slightly different conditions (Kino and Kennedy 1987; Claessens et al. 2017; Dias et al. 2023). Phenotypic divergence from the wild source population is not universally observed (e.g., Reyda and Nickol 2001) but is very common. Among other conditions characteristic of laboratory cultures, improved transmission success to immunosuppressed hosts exerts very different selective pressures than those encountered by wild parasites. What transpired in these laboratory parasite cultures provides a perfect illustration of the rapid and unanticipated evolutionary consequences of even the smallest changes in external conditions.

## 3 The Strength of New Selective Pressures

The Anthropocene represents a *quasi‐permanent directional selection regime*, rather than a fluctuating or reversible selective environment. This may have important implications for parasite evolution, in particular in terms of evolutionary irreversibility or “ratcheting.” And the new selective pressures are strong ones, too.

**FIGURE 1:**
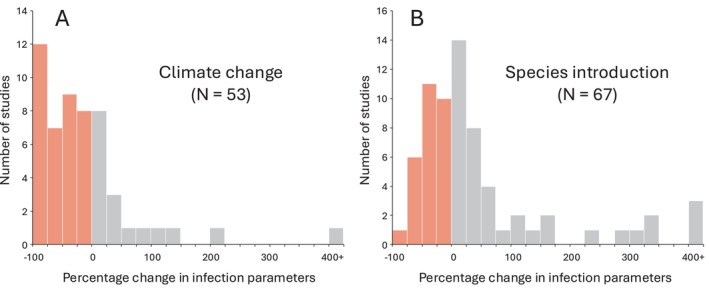
Impact of anthropogenic drivers on parasite populations. Percentage change in population‐level parameters (abundance, incidence, intensity, or prevalence of infection) for eukaryotic parasites in animal hosts, shown separately for two broad categories of anthropogenic impacts: (A) climate change and (B) species introduction. Negative values (pink) indicate a decline in parasite populations relative to their baseline level, whereas positive values (gray) indicate an increase. Estimates were derived from published studies (data from Mahon et al. 2024).

There is now incontrovertible evidence that multiple anthropogenic factors impact the epidemiology of parasites, as seen in positive or negative changes in population‐level parameters such as prevalence or intensity of infection. For example, most types of chemical pollution have some negative effect on the abundance or diversity of aquatic parasites (Blanar et al. 2009). In contrast, the unnaturally high‐density conditions in livestock farming and aquaculture are generally associated with higher parasite abundance than what is observed in wild populations (Johnson et al. 2004). The impacts of other aspects of global change are more variable but often cause major changes in the abundance of parasite populations (Mahon et al. 2024; Wolmuth‐Gordon et al. 2025). For instance, in the majority of host–parasite systems studied to date, the impact of climate change on parasite populations was observed to be negative (68% of cases), whereas that of species introduction was less predictable (Figure 1). Whatever their direction, such population‐level effects are clear indicators that human activities can exert strong selective pressures on parasites.

Anthropogenic factors can also shape individual‐level phenotypic traits associated with parasite fitness, such as stage‐specific survivorship, growth rate, age at maturity, or reproductive strategies (Poulin et al. 2024). For instance, the survival of the free‐living infective stages of parasites can be directly affected by environmental pollutants or climate change (Table 2), whereas that of postinfection and adult stages can be lowered by vaccination and chemotherapy. If hosts themselves experience the effects of external stressors, such as pollutants causing immunosuppression, parasites can indirectly benefit and achieve higher growth rate and reproduction. Higher than natural host densities, such as those seen in livestock production and aquaculture, can facilitate parasite transmission and thus the survival of infective stages (Krkošek 2010; but see Folk et al. 2026). Conversely, decreased host densities resulting from intensive fisheries and habitat destruction can cause Allee effects as well as lower transmission success and the survival of infective stages. The potential impact on parasites of other recently documented changes in host populations, such as loss of genetic diversity (Clark et al. 2025), is difficult to predict. There is no one‐size‐fits‐all scenario: the direction and magnitude of impacts of anthropogenic factors on parasite survival, growth and reproduction depend on the particular factors involved, the host's response, and the parasite taxon and its life cycle. Therefore, there can be no universal evolutionary response of parasites to human activities and the selective pressures they exert; even the same host–parasite systems might respond differently in different local environmental conditions. All we can be sure of is that any sustained change to the age‐dependent mortality rates of parasites and to factors determining their schedule of growth, maturation, and reproduction will inevitably select for phenotypic responses and ensuing genetic changes (Stearns 1992). These can manifest over a few generations as shifts in particular traits, or altered trade‐offs between traits, such as between virulence and parasite lifespan, or the one between egg numbers and egg sizes that captures the range of investment strategies into reproduction.

**TABLE 2:** Selected studies highlighting the difference (percentage) in parasite performance and fitness parameters (i.e., replication, survival of infective stage, survival of adult stage, etc.) between parasites in control conditions versus those exposed to anthropogenic stressors.

Species	Host	Habitat	Anthropogenic stressor	Trait measured	Difference^a^	References
*Schistocephalus solidus* (cestode)	Fish	Freshwater	Temperature (13 vs. 24°C)	Parasite larval growth (weight)	73% increase^b^	Franke et al. (2017)
*Schistocephalus solidus* (cestode)	Fish	Freshwater	Temperature (15 vs. 20°C)	Parasite larval growth (weight)	296% increase	Macnab and Barber (2012)
*Schistocephalus solidus* (cestode)	Fish	Freshwater	Temperature (15 vs. 20°C)	Estimated adult fecundity based on larval size	1530% increase	Macnab and Barber (2012)
*Maritrema poulini* (trematode)	Snail	Freshwater	Temperature (16 vs. 20°C)	Cercarial dispersal	206% increase	Selbach and Poulin (2020)
*Apatemon* sp. (trematode)	Snail	Freshwater	Temperature (16 vs. 20°C)	Cercarial dispersal	77% increase	Selbach and Poulin (2020)
*Galactosomum* sp., *Maritrema novaezealandensis*, *Philophthalmus* sp., *Parorchis* sp. (trematodes)	Snail	Marine	Ocean acidification	Larval (cercarial) longevity	40%–60% reduction	MacLeod and Poulin (2015)
*Philophthalmus* sp. and *Parorchis* sp. (trematodes)	Snail	Marine	Ocean acidification	Metacercarial survival	0%–78% decrease	MacLeod and Poulin (2015)
*Diplostomum* sp. (trematode)	Snail	Freshwater	Pollution	Cercarial survival after 24 h	0%–45% decrease^b^	Pietrock et al. (2001)
*Notocotylus attenuatus* (trematode)	Snail	Freshwater	Pollution (Copper)	Larval (cercarial) production	Up to 100% decrease^b^	Evans (1982)
*Philophthalmus* sp. (trematode)	Snail	Marine	Ocean freshening (35 vs. 30/25 psu)	Larval (cercarial) production	20%–38% decrease^b^	Lei and Poulin (2011)
*Philophthalmus* sp. (trematode)	Snail	Marine	Ocean freshening (35 vs. 30/25 psu)	Encystment success	35%–75% decrease^b^	Lei and Poulin (2011)
*Meloidogyne floridensis* (nematode)	Plant	Terrestrial	Temperature (26 vs. 32°C)	Egg production	133% increase	Sagar et al. (2025)
*Pomphorhynchus laevis* (acanthocephalan)	Crustacean	Freshwater	Temperature (14 vs. 17°C)	Development time of parasite in intermediate host	37% decrease	Labaude et al. (2020)
*Lepeophtheirus salmonis* (copepod)	Fish	Marine	Temperature (5 vs. 20°C)	Number of eggs	55% decrease^b^	Samsing et al. (2016)
*Lepeophtheirus salmonis* (copepod)	Fish	Marine	Temperature (5 vs. 20°C)	Infection window duration	60% decrease^b^	Samsing et al. (2016)
*Lepeophtheirus salmonis* (copepod)	Fish	Marine	Temperature (5 vs. 20°C)	Infection success	1880% increase	Samsing et al. (2016)
*Lepeophtheirus salmonis* (copepod)	Fish	Marine	High host density	Early fecundity	13%–41% decrease^b^	Folk et al. (2026)
*Lepeophtheirus salmonis* (copepod)	Fish	Marine	High host density	Lifetime fecundity	38%–75% decrease^b^	Folk et al. (2026)

^a^ Difference relative to control or baseline values representing non‐stress conditions.

^b^ Values extracted from figures or raw data.

## 4 Types of Evolutionary Responses

If they persist over time and maintain a constant direction, strong selective pressures of anthropogenic origins can result in at least two types of adaptive responses in parasite populations. First, this is most obvious when the mean value of traits under selection displays directional evolution, shifting gradually from generation to generation toward either higher or lower values. Selection will favor individuals at one extreme of the phenotypic distribution if they achieve higher fitness, changing allele frequencies over time and causing the trait distribution to shift toward a new optimum. For example, after 2 years and several generations of regular exposure to anthelmintics and thus lower expected adult lifespan, nematodes (*Teladorsagia circumcincta*) parasitic in sheep evolved faster growth toward a larger adult size compared to nematodes in control populations not exposed to drug treatment (Leignel and Cabaret 2001). Populations of the nematode *Rhabdias pseudosphaerocephala* on the fringes of their amphibian host's expanding geographical range, where host densities are low and the parasite's eggs face a longer wait in the external environment until infection via ingestion, have evolved larger eggs and larvae better equipped for the long wait than those in the centre of the host's range (Kelehear et al. 2012).

The best example comes from the evolutionary changes in the salmon louse, *Lepeophtheirus salmonis* , a copepod ectoparasitic on Atlantic salmon, in response to conditions in fish farms. Extremely high host densities in aquaculture conditions facilitate the parasite's host‐to‐host transmission, and thereby also relax constraints preventing high virulence (Kennedy et al. 2016; Mennerat et al. 2010). In Norwegian salmon farms, salmon lice have not only evolved toward more aggressive feeding on host tissue, that is, greater virulence (Ugelvik et al. 2017), but also toward faster growth and earlier maturation than lice from wild salmon living at much lower densities (Mennerat et al. 2010, 2017). High host densities, by promoting greater exposure to infective stages, may also select for greater resistance to host immune defenses (Folk et al. 2026). Directional selection may result in other salmon lice traits shifting rapidly under aquaculture conditions. For example, the control of salmon lice infections using cleaner fish relies on the visual detection of lice attached to salmon (Barrett et al. 2020), just as the novel laser pulse killing technology requires their detection by a camera, machine vision, and recognition algorithms (Worm et al. 2026). In both cases, these control methods will select for greater transparency or host color‐matching in lice, with individuals whose body coloration shows less contrast with the background host body coloration avoiding detection and surviving longer (Hamre et al. 2021). We may therefore expect a rapid shift in mean body coloration, perhaps also in attachment site toward more protected locations on the host's body, in salmon lice populations exposed to these strong selective pressures. Even if not measurable within months of the introduction of cleaner fish to salmon cages (Imsland et al. 2023), evolutionary changes in lice pigmentation seem inevitable in the longer term. Similarly, freshwater immersion of salmon is also used to control salmon lice, as lice are not tolerant of low salinities. However, this procedure, if repeatedly used, will potentially select for the more resistant genotypes and rapidly shift salinity tolerance in the lice population (Groner et al. 2019).

The second type of adaptive response is less obvious: parasite performance may remain mostly unchanged although external conditions and selective forces are changing. Genotypes with higher tolerance of the new conditions increase in frequency and achieve the same average performance as genotypes that had higher tolerance of original conditions. Thus genotype frequencies change but average phenotype or performance across the population does not; instead, it just shifts over time along some environmental gradient. This is best illustrated using thermal performance curves. These are unimodal and continuous reaction norms capturing the response of a given genotype, in terms of any performance metric related to fitness, across a gradient of temperature (Buckley and Kingsolver 2021). The use of thermal performance curves has been advocated for predicting the impact of climate change on parasitism and diseases (Mordecai et al. 2019; Byers 2020; Rohr and Cohen 2020). These curves have a nearly universal asymmetric shape across all ectotherms, with performance rising almost exponentially with increasing temperature toward a peak value at the optimal temperature and falling steeply toward zero with further temperature increases (Arnoldi et al. 2025). Under a sustained global warming scenario, individuals whose peak performance is attained at a slightly higher temperature will be favored generation after generation, resulting in a gradual shift of the population's average thermal performance curve toward higher temperatures, but not necessarily with a change in the mean value of the trait. One has to plot the performance curves of different genotypes to be able to identify differences in optima (the actual trait) and to trace shifts in response to selective pressures over time. Hence, the absence of easily detectable phenotypic differences between pre‐ and post‐selection individuals does not mean the absence of evolution.

The asexual reproduction of trematodes within their intermediate hosts and its sensitivity to temperature provides an excellent example. In their molluscan first host, trematodes multiply asexually to produce huge numbers of genetically identical infective stages (cercariae), which leave the mollusk to seek the next host in their life cycle. Numerous experimental studies have shown that the production of cercariae increases rapidly with temperature, up to a point beyond which it drops abruptly toward zero (e.g., Morley and Lewis 2013; Khosravi et al. 2023; Stout et al. 2025). With gradual increases in ambient temperature, we might expect that genotypes whose peak rate of within‐host multiplication coincides with slightly higher temperatures will be at an advantage. The average thermal performance curve of the population would then be slowly displaced toward higher temperatures. Without changes in the availability of host resources and all else being equal, future trematodes will not produce more cercariae because it is warmer; they will just evolve to produce the same amount in the new thermal environment. The unchanging production level is the consequence of a temporal shift in a key trait—temperature tolerance—of the parasite genotypes. Should this be the case, earlier predictions that global warming will result in greater output of infective cercariae and elevated infection risk for other hosts in the trematode life cycle (Poulin 2006; Mas‐Coma et al. 2009) may not materialize. Shifting performance curves, whether as a function of temperature or any other factor, in this and other cases are only one of several possible scenarios, of course, but they illustrate a type of adaptive response that must be considered in predictive modeling of disease risk under climate change.

## 5 Monitoring Rapid Parasite Evolution

Even if rapid parasite evolution should be expected when new and strong selection pressures arise, tracking its speed and direction is essential in order to predict evolutionary changes and take prompt action to either remedy their consequences or compensate for them. Controlled experiments in which parasites are subject to artificial selection or exposed to novel conditions across multiple generations are informative (see Table 1). However, monitoring parasite evolution in real time, in the real world, is what we really need. Tracking “natural” experiments where we already know parasites are rapidly evolving under strong directional selection, such as mass anthelmintic usage in livestock or crowded rearing conditions in fish farming, achieves exactly this (Skorping and Read 1998; Leignel and Cabaret 2001). So would snapshot resampling of well‐studied populations, or long‐term studies of selected host–parasite systems exposed to anthropogenic stressors in the wild (Hammoud et al. 2026). But how to detect right now or anticipate parasite evolution in situations where it is unclear whether it is even happening, or what traits are under new selection?

To achieve this, one approach is to test retrospectively whether it has happened in the recent past, in parasite populations known to have experienced conditions altered by human activities. There are at least three ways to do this. First, one can use phylodynamics to trace evolutionary change in parasite populations under strong environmental pressures (Grenfell et al. 2004). For instance, data on parasite infection phenotypes can be combined with phylogenetic information and historical environmental data to shed light on recent adaptive shifts. Second, the phenotype of parasites from modern samples could be compared to that of historical samples, from museum or other collections, obtained from the same population, to determine whether changes have occurred after the onset of anthropogenic impacts. This approach is now commonly used to test for human‐induced ecological changes over time, that is, in parasite abundance or diversity (Wood et al. 2023), so why not use it to test for evolutionary changes? In some cases, using dormant parasite infective stages preserved in lake sediment can also allow cross‐temporal infection experiments, to test the performance of historical versus contemporary parasite genotypes (Decaestecker et al. 2007). Such before‐and‐after comparisons can also extend to genetic changes, if DNA can be obtained from historical museum or paleoparasitological samples (Holmes et al. 2016). Third, recent and rapid selection often leaves behind a genomic footprint. For instance, when alleles at one or a few loci, with moderate to strong phenotypic effects, sweep through a population toward high frequencies or even fixation under strong selection, they leave a trace (Messer and Petrov 2013; Stephan 2016). Genome scanning approaches can detect selective sweeps via altered frequency spectra of alleles flanking the positively selected loci which result from linkage disequilibrium (Abondio et al. 2022). Such genomic signatures of recent, rapid evolution in living parasites would be telltale signs that they have responded to novel selection pressures.

Another promising approach that may serve to predict what evolutionary changes to expect in the future involves space‐for‐time contrasts. Here, comparisons among populations exposed to varying degrees of a particular anthropogenic pressure are used instead of comparisons among different time points in the same population experiencing that same pressure (Blois et al. 2013; Lovell et al. 2023). For example, some parasite populations of a given species experience greater levels of heavy metal pollution than others, or their habitat has been modified to different degrees by habitat destruction or species introductions. Phenotypic and genetic divergence among these populations can reflect local adaptation to local conditions, and they can be used to predict what would happen in a single population undergoing directional evolution driven by a particular anthropogenic pressure. The above methods provide us with a diverse set of tools to detect, monitor, and anticipate rapid parasite evolution in the Anthropocene.

## 6 Mitigation Strategies?

**FIGURE 2:**
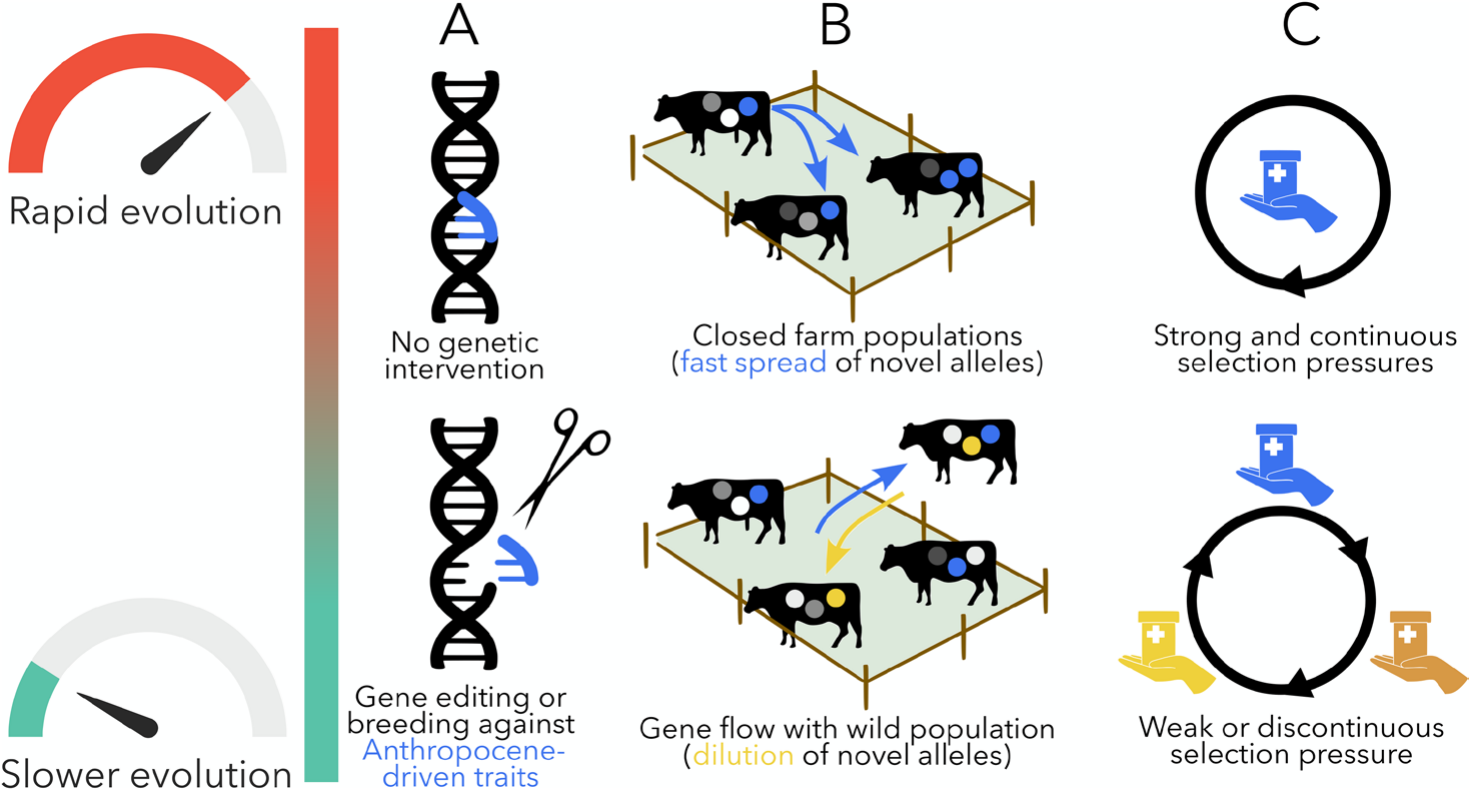
Mitigation of rapid parasite evolution in the Anthropocene. Examples of possible strategies to reduce the rapid spread of parasite adaptations in the typical selective landscape of livestock farming and aquaculture. These include (A) genetic manipulations or selective breeding approaches, (B) allowing gene flow between domesticated and wild parasite populations to dilute the spread of unwanted traits, and (C) discontinuity in selection pressures (illustrated here as alternation of anti‐parasite drug types). See main text for further examples.

There is only one way of ending, or at least easing, the selective pressures acting on parasites and other organisms that cause them to rapidly evolve: removing the source of pressure. That is easier said than done. Mounting evidence has failed to convince governments and industries to take the necessary steps to act on global climate change and other environmental damage. For all organisms in natural systems, the selective pressures will therefore remain. Mitigation is therefore our only hope, and here we present a first attempt to outline general strategies to combat rapid parasite evolution (Figure 2) and discuss approaches toward their practical implementation.

Although natural ecosystems may be out of reach, in production systems certain selective pressures can be controlled, at least in principle. For livestock farming and aquaculture, the ultimate goal will remain the elimination of parasites. Since that goal is out of reach at present, controlling the abundance and impacts of parasites has been the industry's main focus. Rotating anthelmintics, that is, alternating the chemical classes of drugs used to treat animals against parasites, has long been seen as a strategy to slow down the evolution of parasite resistance to any particular drug (Coles and Roush 1992). This approach interrupts the otherwise continuous selection pressure acting on parasites to evolve adaptations against one drug's mechanism of action. For ectoparasites such as salmon lice, rotating the physical treatments (hydrogen peroxide, hot water baths, freshwater exposure, mechanical removal) used to delouse fish would also slow down the evolution of any anti‐treatment adaptations in the parasite population, avoiding what happened with chemotherapy (Besnier et al. 2014). Apart from chemotherapy and physical treatments, selective pressures arise from the other unnatural conditions experienced by parasites in livestock farming and aquaculture: they exploit a high‐density host population consisting of genetically homogeneous individuals. These conditions enhance parasite transmission success, lower the mortality usually incurred by infective stages, and select for different combinations of parasite life history traits and different levels of virulence (Leignel and Cabaret 2001; Mennerat et al. 2010, 2017; Kennedy et al. 2016; Ugelvik et al. 2017). Several farming practices used primarily to reduce infection levels can also indirectly slow down or prevent rapid parasite evolution. For example, rotational grazing, whereby livestock are regularly moved to a new paddock, can interrupt the parasites' life cycle and raise the mortality of infective stages to levels closer to natural ones (Bransby 1993). Any strategy that reduces the actual or effective density of hosts will have the same effect. For instance, mixed‐species grazing lowers the density of each host species relative to what it would be in a monospecific system (Forteau et al. 2020). This results in the infective stages of parasites specific to one host species often reaching a dead end in the other host species and increasing their mortality rate. Similar practices would also work in an aquaculture context. It is therefore possible to exercise some control over the selective pressures acting on parasite populations in animal production systems and sidestep their evolutionary consequences.

Another possible mitigation strategy would be to avoid the genetic isolation of parasite populations that are under particularly strong selective pressures (e.g., in aquaculture or livestock farming) where certain traits can be rapidly selected (fast reproduction, drug resistance, high virulence). By actively promoting genetic exchanges between these populations and those under more natural conditions, we might offset the rapid evolution of undesirable traits. Gene flow between livestock parasites and those infecting wild ungulates is often seen as driving allele introgression by introducing maladaptive traits and reducing viability in wild parasite populations, with traits such as drug resistance carrying a fitness cost in the absence of the drug (Redman et al. 2012; King et al. 2015). There are also concerns about the possible role of wild host populations acting as vectors, reservoirs and/or refuges for drug‐resistant genotypes (Chintoan‐Uta et al. 2014; Beaumelle et al. 2024). Yet, there is an alternative scenario: parasite gene flow between farmed and wild host populations could “dilute” rare alleles favored by anthropogenic pressures and slow down the evolution of undesirable parasite traits. This possibility deserves further research.

Other approaches can also be envisaged to control the evolution of parasites in these systems and steer it in desirable directions. Genome editing offers one possible avenue. The biased inheritance of specific genes and their spread throughout a wild population are theoretically possible through engineered gene drives (Esvelt et al. 2014; Champer et al. 2016). This might allow partial control over the evolution of parasite traits such as virulence, growth rates, or egg production. If eradication of economically damaging parasites proves impossible through this approach or another, genome editing might at least allow their replacement by more benign variants. This would require breeding genetically modified parasites in experimental hosts before releasing them in the real world. Of course, releasing genetically modified parasites into livestock or aquaculture systems may have unforeseen consequences; therefore, the potential benefits would need to be carefully weighed against the potential risks. How applicable these new technologies are for parasites of livestock and farmed fish remains to be seen; however, they represent intriguing possibilities to counter human‐induced parasite evolution. Furthermore, any strategy to mitigate rapid parasite adaptation in controlled animal populations may also benefit wild host populations, given frequent spillover events and other parasite exchanges from the former to the latter.

## 7 Conclusion

By definition, parasites impact the physiology, health, behavior, and survivorship of individual animals. They have long been a source of disease for humans and domesticated animals and have caused major economic losses. In natural systems, they also modulate the dynamics of host populations (Hudson et al. 1998; Watson 2013), influence the diversity of communities (Wood et al. 2007; Friesen et al. 2020), affect the complexity and stability of food webs (Lafferty et al. 2008; Hatcher et al. 2012), and their biomass often exceeds what meets the eye to the point of impacting ecosystem energetics (Kuris et al. 2008; Preston et al. 2013). Our activities are not only changing the magnitude of these ecological impacts; they are also changing the parasites themselves.

The short‐term responses of parasite transmission and abundance to anthropogenic stressors are idiosyncratic: they are contingent upon the taxa involved, the frequency and/or strength of the novel stressor, and other external factors (Blanar et al. 2009; Mahon et al. 2024; Wolmuth‐Gordon et al. 2025). This, in itself, greatly complicates attempts at predicting future parasite abundance and disease risk as we move forward in the Anthropocene. The fact that parasites are evolving rapidly, however, represents a greater problem for future forecasts. The empirical data used to assess how parasites will respond to anthropogenic stressors come mostly from experimental studies in which today's parasites are exposed to tomorrow's conditions. However, regardless of how informative these studies are, they suffer from an inescapable flaw: the parasites that will actually experience future conditions are tomorrow's parasites, not today's. The challenge for anyone attempting to predict disease risk in a changed world will be to incorporate parasite evolution into models to produce more realistic scenarios that account for likely changes in parasite phenotypes.

The main task at present remains to better understand parasite evolution in our rapidly changing world: what traits are being selected, in what direction, under what conditions, and how fast are they changing. We only have partial answers to these questions, for only a small handful of host–parasite systems. Beyond the evolution of drug resistance, the rapid evolution of parasites is not widely appreciated, yet it threatens food production systems, ecosystem health, and human well‐being in a One Health perspective. Our hope is that this warning will serve to mobilize efforts toward broader and more thorough investigations of rapid parasite evolution under Anthropocene conditions, its consequences, and its mitigation.

## Funding

F. Thomas is supported by the Centre National de la Recherche Scientifique and the Camargue Health–Environment Zone Atelier (ZACAM, LTSER‐RZA).

## Conflicts of Interest

The authors declare no conflicts of interest.

## Data Availability

Data sharing is not applicable to this article as no datasets were generated or analyzed during this study.
